# Comparative Analysis of Pyrosequencing and a Phylogenetic Microarray for Exploring Microbial Community Structures in the Human Distal Intestine

**DOI:** 10.1371/journal.pone.0006669

**Published:** 2009-08-20

**Authors:** Marcus J. Claesson, Orla O'Sullivan, Qiong Wang, Janne Nikkilä, Julian R. Marchesi, Hauke Smidt, Willem M. de Vos, R. Paul Ross, Paul W. O'Toole

**Affiliations:** 1 Department of Microbiology, University College Cork, Cork, Ireland; 2 Alimentary Pharmabiotic Centre, University College Cork, Cork, Ireland; 3 Teagasc, Moorepark Food Research Centre, Moorepark, Fermoy, Co. Cork, Ireland; 4 Center for Microbial Ecology and Department of Microbiology and Molecular Genetics, Michigan State University, East Lansing, Michigan, United States of America; 5 Department of Basic Veterinary Medicine, Division of Microbiology and Epidemiology, University of Helsinki, Helsinki, Finland; 6 Laboratory of Microbiology, Wageningen University, Wageningen, The Netherlands; University of Hyderabad, India

## Abstract

**Background:**

Variations in the composition of the human intestinal microbiota are linked to diverse health conditions. High-throughput molecular technologies have recently elucidated microbial community structure at much higher resolution than was previously possible. Here we compare two such methods, pyrosequencing and a phylogenetic array, and evaluate classifications based on two variable 16S rRNA gene regions.

**Methods and Findings:**

Over 1.75 million amplicon sequences were generated from the V4 and V6 regions of 16S rRNA genes in bacterial DNA extracted from four fecal samples of elderly individuals. The phylotype richness, for individual samples, was 1,400–1,800 for V4 reads and 12,500 for V6 reads, and 5,200 unique phylotypes when combining V4 reads from all samples. The RDP-classifier was more efficient for the V4 than for the far less conserved and shorter V6 region, but differences in community structure also affected efficiency. Even when analyzing only 20% of the reads, the majority of the microbial diversity was captured in two samples tested. DNA from the four samples was hybridized against the Human Intestinal Tract (HIT) Chip, a phylogenetic microarray for community profiling. Comparison of clustering of genus counts from pyrosequencing and HITChip data revealed highly similar profiles. Furthermore, correlations of sequence abundance and hybridization signal intensities were very high for lower-order ranks, but lower at family-level, which was probably due to ambiguous taxonomic groupings.

**Conclusions:**

The RDP-classifier consistently assigned most V4 sequences from human intestinal samples down to genus-level with good accuracy and speed. This is the deepest sequencing of single gastrointestinal samples reported to date, but microbial richness levels have still not leveled out. A majority of these diversities can also be captured with five times lower sampling-depth. HITChip hybridizations and resulting community profiles correlate well with pyrosequencing-based compositions, especially for lower-order ranks, indicating high robustness of both approaches. However, incompatible grouping schemes make exact comparison difficult.

## Introduction

The intestinal microbiota has an important role in maintaining health throughout mammalian lives [1]. Although many studies have focused on how microbial communities are structured during the early and middle stages of life, relatively little is known about gut microbiota of the elderly. For instance, there have been reports on decreased microbial diversity in general [2], [3], and depletion of beneficial bacteria such as bifidobacteria in particular [4], although these trends have not been universally reported [5], [6]. These studies have previously been supported by quantitative analysis of the ubiquitous microbial 16S ribosomal RNA gene using traditional molecular methods like denaturing gradient gel electrophoresis (DGGE), fluorescent *in situ* hybridization (FISH), quantitative PCR (qPCR), or capillary sequencing using the Sanger method [7]. However, for a complex and microbe-dense ecosystem like the human gut, these methods provide an incomplete view of the microbial composition, revealing only the most abundant taxa. In a meta-analysis by Rajilic'-Stojanovic' and colleagues [8], almost 1,200 phylotypes were identified based on 98% sequence similarity cut-off of full-length SSU rRNA sequences, with an estimated total richness of over 3,000 phylotypes. In recent years, the rapid development of next-generation sequencing technologies has allowed vast numbers of partial 16S rRNA genes from uncultured bacteria to be sequenced, at a much lower cost than Sanger dideoxy sequencing. In addition to bypassing previously needed cloning and/or cultivation procedures, with their associated biases, community structures can now be investigated at much higher resolution by revealing taxa that are much less abundant. However, this may be at the expense of lower taxonomic certainty due to the shorter read lengths of sometimes poorer quality.

Recent high-throughput microbial compositional studies have used the pyrosequencing technology introduced by 454 Life Science [9], whereby amplicons of partial 16S rRNA gene sequences are attached and sequenced on microscopic beads placed separately in picoliter-sized wells. For the Genome Sequencer 454 FLX system, this generally produces around 400,000 reads with average lengths of 250 bp and an average quality score of greater than 99.5% accuracy rate [10]. These read sizes are sufficient to cover most of the variable regions in the 16S rRNA gene. A large number of samples can be pooled onto one plate by including short barcode sequences, or multiplex identifiers (MIDs), upstream of the PCR primers specific for the variable region to be sequenced. Pyrosequencing has been applied to a wide range of microbial communities and variable regions of the 16S rRNA gene, such as V6 in deep-sea vents microbial communities [11], [12]; V1, V2, V6 and V3 in human [13]–[16] and in macaque [17] gastrointestinal tract (GIT); as well as V9 in soil-derived microbial DNA [18].

High-throughput community analyses do not have to depend on sequencing. A number of phylogenetic arrays have been constructed that permit hybridization of nucleic acids extracted from environmental samples against arrays probes corresponding to single-stranded full or partial 16S rRNA genes [19]–[22]. As it is technically very difficult to include the more than 800,000 SSU sequences present in the databases (see http://www.arb-silva.de), microarrays with subsets of sequences specific to the ecological environment of interest are required. Recently the HITChip, an oligonucleotide microarray for phylogenetic profiling of human intestinal tract communities, was developed [23]. The 4,800 probes on this 16S rRNA gene tiling array consist of sets of three 18–30 nt long overlapping oligonucleotides targeting the V1 and V6 region sequences from 1,140 phylotypes, respectively. Based on 98% sequence similarity, phylotypes were defined from more than 16,000 16S rRNA gene sequences identified in the human GIT. Using the HITChip for comparing phylogenetic profiles of fecal microbiota from five young and five elderly adults collected at three time points, Rajilic'-Stojanovic' and colleagues confirmed previous findings that the adult fecal microbiota is highly individual-specific and relatively stable over time [24]–[26]. With the aid of this technology it was also shown that a multispecies probiotic cocktail alleviated symptoms of irritable bowel syndrome [27], and that starch-fermenting bacteria could be identified by using RNA stable isotope probing in a human colon model with great reproducibility [28]. However, when compared to high-throughput sequencing, phylogenetic arrays can only detect taxa that are covered by the reference sequences. In addition, the dynamic range of detection is smaller, and cross-hybridization between probes may occur. There are also fewer options for downstream analysis compared to ribosomal sequences. On the other hand, arrays are more straight-forward to use for comparative community profiling, and are generally both faster and cheaper than high-coverage amplicon sequencing. A comparison of microarray hybridization and sequencing of 16S rRNA gene clone libraries was conducted by Palmer and colleagues, and showed strong concordance between the two methods [20]. However, a later study highlighted the poor resolution of clone library sequencing in relation to microarray profiling [29]. This raised the question of how phylogenetic array analysis compares with deep pyrosequencing, which was one of the main objectives of this study.

A crucial part of community analysis is the classification of sequences into a taxonomic framework. A diverse range of methods has been used, with dramatic differences in classification results depending on both underlying algorithms and parameters. Due to the requirement for large datasets, classification methods based on parsimony and likelihood trees typically applied on Sanger-sequenced full-length 16S rRNA genes are not feasible. Liu and colleagues [30] assessed some of the most commonly used methodologies for a number of different variable regions within the 16S rRNA gene. These methods included *i)* selecting the most common classification from the best BLAST [31] hits against reference sequences from the RDP database; *ii)* the online RDP-classifier with bootstrap values≥50% (see further below); *iii)* the online Greengenes classifier [32] based on NAST alignments [33]; *iv)* selecting the nearest ancestral node in a phylogenetic neighbor-joining tree [34] (similar to the parsimony insertion procedure in ARB, which however is not designed for large numbers of short sequences) built from either *v)* NAST alignments; or *vi)* a distance matrix containing counts of multimers found between sequences. The Greengenes and RDP-classifier produced the most accurate and stable results, especially for gut communities and gave sufficient evidence to support taxonomic classifications [30]. Furthermore, the RDP-classifier is more than 30 times faster than the Greengenes classifier and is also available as a downloadable version [30]. SSU rRNA gene fragments of at least 250 bp covering the V2, V3 and V4 regions were deemed to be the most suitable. In contrast, the hyper-variable V6 was shown to be the least optimal region for taxonomy assignments, while it was more appropriate for measuring microbial diversity due to its high variability. In addition, three other comparative studies favored V1, V2 and V4 based on BLAT (original software ref. [35]) searches against the RDP database [36], and V2 and V4 based on the RDP-classifier [37], as well as V2 and V3 based on ClustalW (original software ref. [38]) alignments and Neighbor-joining trees [39]. The assignment tool GAST was recently reported [40], which uses the best BLAST hits against a reference database of V3 and V6 regions where the taxonomy is known from RDP-classification results. Instead of selecting the most common classification of these hits (like in method *i* above), the sequence was assigned to the hit that had the smallest global distance in a distance matrix based on MUSCLE [41] alignments of the best hits. Using GAST, more than 99% of V3 and V6 sequences could be assigned to taxa at the genus level.

This study is a comparison of two high-throughput molecular methods [36], [37] for GIT community analysis using subjects exclusively from the elderly population. To achieve the desired high coverage necessary for this study, we had to limit the number of regions targeted for pyrosequencing. Thus, the two variable regions that were targeted for pyrosequencing in the present study were V4, for documented classification robustness (see references above); and V6, for hyper-variability and number of published studies. Moreover, for assigning taxonomies we chose the RDP-classifier due to its documented accuracy and stability, straight-forward usage, independence of sequence alignments, high speed, and suitability for very large datasets generated by next-generation sequencing technologies. The classifier is also integrated with the Pyrosequencing Pipeline [42] which contains tools for quality trimming, in-depth comparison of two communities, phylotype clustering, as well as statistical and ecological metrics like rarefaction curves, diversity index and richness estimations. In contrast to nearest-neighbor methods, the RDP-classifier bases its assignments on the probability of observing a set of eight-character subsequences from an unknown query sequence within each genus. The RDP-classifier is trained with more than 7,000 bacterial full-length SSU rRNA sequences, composed mostly of sequences from type strains. Confidence estimations are also generated for each assignment, representing the number of times the assigned taxa was selected out of 100 bootstrap trials [37].

In this study, we sequenced regions of the 16S rRNA gene at very high depth for a small number of samples (four), resulting in a majority of the estimated GIT microbial diversity being captured. We also noticed considerable differences between the two variable regions V4 and V6, both in terms of classification efficiency and captured diversity. The robustness of the HITChip and RDP-classifications of pyrosequencing reads were supported by their strong correlations at several taxonomic levels. In addition to providing useful comparisons of high-throughput technologies, variable regions and analysis protocols, this analysis acts as a pilot study for validating methodologies for a large-scale national metagenomics initiative (see http://eldermet.ucc.ie), by defining how much sequencing is necessary to sufficiently capture the community diversity at an affordable depth of sampling.

## Results and Discussion

### Quantitative compositional sequence analysis

We sequenced a total of 1,668,550 variable regions of the 16S rRNA gene, amplified from microbial DNA extracted from fecal samples from elderly individuals coded A, B, C and D. Because different levels of pyrosequencing coverage were applied, we indicate this by a suffix referring to the proportion of the picoliter plate that was dedicated to each sample. Of the total number of reads, 807,953 were of the V6 region from samples A and B (designated A-V6-1.0 and B-V6-1.0), and 860,597 were half-plate runs of the V4 region from samples A, B, C and D (A/B/C/D-V4-0.5). In addition, V4 amplicons from the C and D samples were sequenced at a lower depth on another plate (42,315 and 40,741 reads, respectively) as part of the larger group of subjects being analyzed by the full-scale Eldermet project, and are thus referred to as C-V4-0.1 and D-V4-0.1. Quality filtering removed 14% of V4 and 35% of V6 sequences (see Table 1 for details of numbers). The pyrosequencing artifact of technical read duplications highlighted by Turnbaugh and co-workers [15] was not an issue here; at most 0.05% of all reads among the four pyrosequencing plates, (pooled sample C and D) had more than one copy with the exact same length, content and quality scores. Biological duplications, however, were as expected much more common; 18% of the A-V6-1.0 reads represented unique sequences, while 26.8% for A-V4-0.5. The quality-trimmed V4 and V6 reads had an average length of 224 bp and 79 bp, respectively.

**Table 1:** Statistical characteristics of V4 and V6 amplicon sequence analysis from the four fecal samples, at two different similarity levels.

Datasets	ALL-V4		A-V4-0.5		B-V4-0.5		A-V6-1.0		B-V6-1.0		C-V4-0.5		D-V4-0.5		C-V4-0.1		D-V4-0.1	
**Untrimmed reads**	860,597		213,294		235,489		402,823		405,130		206,365		205,449		47,100		44,907	
**Trimmed reads**	740,704		185,003		194,316		267,104		260,042		176,106		185,279		42,315		40,741	
**Similarity**	97%	98%	97%	98%	97%	98%	97%	98%	97%	98%	97%	98%	97%	98%	97%	98%	97%	98%
**Phylotype richness**	5,249	11,623	1,686	3,621	1,472	3,074	12,049	14,533	12,545	14,738	1,629	3,701	1,778	3,467	574	1,103	678	1,186
**Chao1 richness estimation**	7,847	17,408	2,584	5,310	1,977	4,217	18,950	25,993	19,045	25,976	2,461	5,286	2,675	5,300	931	1,655	1,000	1,805
**Chao1-LCI95**	7,563	16,942	2,415	5,076	1,868	4,038	18,466	25,281	20,027	25,293	2,302	5,062	2,513	5,045	828	1,528	913	1,671
**Chao1-UCI95**	8,165	17,915	2,793	5,581	2,115	4,429	19,470	26,752	19,470	26,704	2,657	5,548	2,872	5,597	1,077	1,818	1,120	1,975
**Shannon diversity**	6.400	8.105	4.913	5.678	4.864	5.678	7.004	7.108	7.038	7.123	4.365	5.413	3.743	4.496	3.626	4.389	3.344	4.178
**Species evenness**	0.747	0.866	0.661	0.693	0.667	0.707	0.745	0.742	0.746	0.742	0.590	0.659	0.500	0.552	0.571	0.626	0.513	0.590
**Good's coverage**	99.72%	99.45%	99.65%	99.31%	99.76%	99.51%	98.00%	97.28%	97.83%	97.12%	99.69%	99.41%	99.62%	99.31%	99.42%	99.01%	99.33%	98.83%

The RDP-classifier assigns taxonomies down to genus level accompanied with bootstrap-like confidence values [37]. As the choice of threshold for these bootstrap values has a significant influence on the outcomes of subsequent analysis, we compared two previously implemented confidence value thresholds of 50% [30] and 80% [40] using the reference set of 7,208 near full-length 16S rRNA genes from human fecal microbiota sequenced by Dethlefsen and colleagues [14]. They compared pyrosequencing reads of the V3 and V6 regions to full-length 16S rRNA sequences from clone libraries from the same samples. We assigned genus to 6,054 sequences using the RDP-classifier with the stricter 80% cut-off and the latest RDP training set 4 from December 2008. With these full-length assignments as references the V3, V4 and V6 regions were extracted and re-classified. Table 2 shows the fractions of variable regions that were classified in accordance with their full-length references, using bootstrap thresholds of 0%, 50% and 80%. The V4 region displayed the highest number (5,091) of correctly classified sequences (according to≥80% classification of full-length sequences) followed by V3 and V6 when using a bootstrap threshold of 80%. The drop in accuracy, when decreasing the threshold to 50%, was also the smallest for V4, by which an additional 550 sequences could be classified (97% of all genus-assigned full-length sequences). We therefore decided to use 50% as bootstrap cut-off since the accuracy is closest to the one with 80% cut-off, and the total number of sequences that could be assigned to genus level was closest to that obtained without any cut-off threshold imposed. In terms of absolute numbers of correctly classified reads, V4 is better than V3, which in turn is better than V6. Moreover, the 50% bootstrap value was the chosen threshold for another comparison study promoting the use of the RDP-classifier [30].

**Table 2:** Fractions of variable regions that were correctly classified by the RDP-classifier.

Variable region	V3			V6			V4		
**Bootstrap cutoff (≥)**	0%	50%	80%	0%	50%	80%	0%	50%	80%
**Fraction of sequences classified to genus**	100%	92.4%	82.3%	100%	73.5%	40.4%	100%	97.0%	87.9%
Fraction of sequences correctly classified to genus	92.0%	95.0%	98.1%	79.0%	96.5%	98.7%	92.8%	94.5%	95.7%

Of 7,208 full-length 16S reference sequences from the human gut 6,054 were classified at genus-level with 80% bootstrap support. The RDP-classifier was trained with the latest training set No. 4 from December 2008. For each of the three extracted variable regions fragments were classified again, at three different bootstrap thresholds, and compared with the full-length classifications (last row).

**Figure 1:**
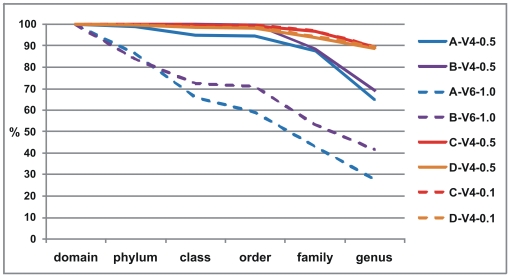
Classification efficiencies at six taxonomic ranks for eight sets of sequences from four samples. The blue and purple colored dashed lines represent V6 amplicon reads, which have very poor classification efficiencies compared to all V4 amplicon reads, especially at the genus level. The yellow and orange colored dashed lines, representing V4-0.1 amplicon reads, show nearly identical classification efficiencies as the corresponding V4-0.5 amplicon reads.

The number of reads that could be classified with a bootstrap value of 50% to a certain taxonomic rank fell as the order of the rank progressed towards genus-level (Figure 1). Interestingly, the classification efficiency for the shorter V6 reads fell dramatically at phylum level relative to the V4 reads, and decreased further to below 50% at genus level. Possible reasons for this are: *i)* the much shorter V6 lengths; *ii)* that its hyper-variable and poorly conserved sequence impedes high-confidence classifications; and/or *iii)* that it is flanked on both sides with highly conserved sequences which add little classification information. Although the corresponding numbers for the V4 reads were much higher, there were significant differences at the genus level between samples A and B (65–70%), and samples C and D (∼89%). Following closer inspection, we found that a majority (∼60%) of the differences in these ratios were due to the higher numbers of unclassified genera within the *Lachnospiraceae* family in samples A and B, pointing towards the need for a more rigorous taxonomic classification within phyla largely dominated by yet uncultured phylotypes, such as is the case for the *Lachnospiraceae* [8]. Another explanation may be that fewer reads have been confidently classified as phylum *Bacteroidetes* in samples A and B; the average genus bootstrap values for the two most numerous phyla were found to be 93% for *Bacteroidetes* and 71% for *Firmicutes*. The lower *Bacteroidetes* counts in samples A and B may be due to differential cell lysis of bacteria belonging to this phylum imposed by premature freezing and further processing of these fecal samples. This unexpected lack of *Bacteroidetes* has also been recorded in other studies, where fecal samples have been frozen immediately upon collection [13], [17], [43]. Moreover, it has been shown that DNA extraction protocols affect the isolation of *Bacteroidetes* is the subject of a separate systematic study (Salonen, de Vos *et al.,* in preparation). Overall, these examples illustrate the significant impact that the overall community structure can have on the ability to classify large fractions of its members, even if the type of environment is the same.

**Figure 2:**
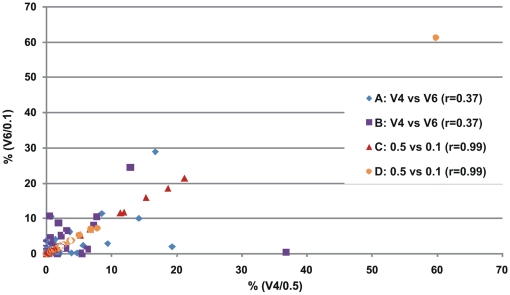
Pearson correlations between genus-classifications for V4 and V6 amplicon sequence datasets, as well as C-V4-0.5 and D-V4-0.5, and C-V4-0.1 and D-V4-0.1 samples.

The classification efficiencies of the C/D-V4-0.1 samples were practically identical to the corresponding samples sequenced 4–5 times deeper. This was also supported by the 99.99% Pearson correlation between the genus classifications of C-V4-0.5 and D-V4-0.5, and C-V4-0.1 and D-V4-0.1 (Figure 2). In contrast, correlations between genus classifications for the V4 and V6 amplicon sequences were very poor (A: 69% and B: 37%), which can be attributed to the inferior ability to classify V6 reads at genus level, in particular those belonging to the *Lachnospiraceae Incertae Sedis*.

**Figure 3:**
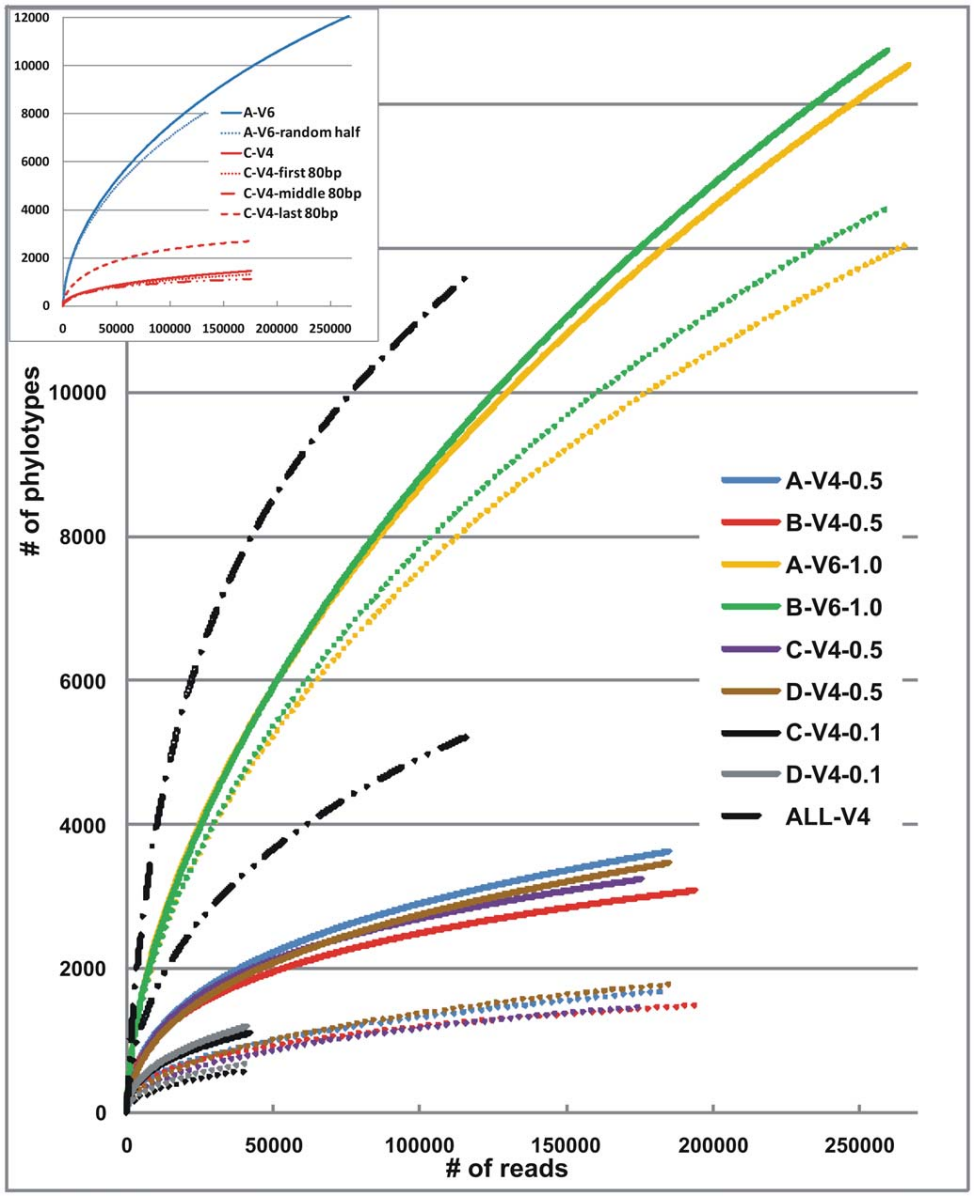
Rarefaction curves at 97% (dotted lines) and 98% levels (solid lines, except for ALL-V4 which has single dots) for all eight datasets including a combination of all V4-0.5/0.1 datasets sequences. The inset also shows curves for half the A-V6-1.0 reads and the three constituent parts of the C-V4-0.5 reads.

Determining community composition based upon a highly variable SSU region would indicate greater apparent community complexity (reflected in phylotype number) than would a less variable region [23]. To measure how phylotype richness in the four fecal communities varied with sample size and choice of variable region, we calculated rarefaction curves at both 97% and 98% similarity levels (Figure 3). Richness levels measured by the V6 region vastly exceeded those using the V4 region, with 4–5 times more identified phylotypes at the 98% level and 7–9 times more phylotypes at the 97% level (Table 1). To verify that this huge difference was due to higher variability within the V6 region, and not relative oversampling, the rarefaction curves were re-created using half of the A-V6-1.0 reads (randomly selected), as well as three constituent tracts of the C-V4-0.5 region reads (Figure 3 inset). Although the last 80 bp of the V4 region displays slightly higher variability than the downstream parts, it is clear that, even at lower number of sequenced reads, the V6 region is far less conserved than V4.

As this is the deepest sequencing analysis imposed to date, upon individual-derived GIT communities, we discovered, as expected, the highest number of phylotypes in a single sample using both variable regions (Table 1; for easier comparison with other studies we only discuss phylotypes defined by 97% similarity below). More than 12,500 V6 phylotypes were identified in sample B, and almost 1,800 V4 phylotypes in D. According to Chao1 richness estimations that were supported by rarefaction curve extrapolations, these communities contain 500–1,000 additional phylotypes using V4, and 6,500 more using V6, with final richness roughly around 2,500 and 19,000, respectively. Hence, even at this high level of sequencing it is evident that additional sampling increases the number of phylotypes detected. When all V4 sequences (740,704 trimmed reads) from the four samples were pooled together, more than 5,200 phylotypes were observed at the 97% similarity level, which is higher than any previously reported richness at that level (notably using different variable regions and datasets). Interestingly, at the same similarity level, McKenna and co-authors also detected about 5,000 phylotypes condensed from about 141,000 pyrosequencing reads of concatenated V1 and V2 regions from 100 GIT samples collected from 12 macaques [17]. Moreover, Ley and colleagues identified close to 4,700 unique phylotypes (at≥96% similarity) from over 20,000 full-length 16S rRNA genes sequenced from 60 mammalian species [44]. If this is an indication that we have successfully detected the majority of the total number of phylotypes within mammalian fecal communities, Chao1 richness estimation and extrapolation of the ALL-V4 rarefaction curve suggests a total richness level of around 8,000 phylotypes. Future large-scale studies including many more subjects will show if this is correct. The fact that the under-sampling of the C-V4-0.1 and D-V4-0.1 communities revealed fewer phylotypes than their full-sample-size correspondents at the same sampling level (∼40,000 reads) highlights the imperfect and overestimating effect of sub-sampling within rarefaction. Moreover, Chao richness estimations of the C/D-V4-0.1 communities are 62% lower than for C/D-V4-0.5. This indicates an underestimating effect for less sampled communities, which has also been observed [14], [45] and discussed [46], [47] by others.

Good's coverage is an estimator of sampling completeness and calculates the probability that a randomly selected amplicon sequence from a sample has already been sequenced. At the 97% similarity level, all four V4-0.5 samplings had more than 99.6% coverage, which means that over 250 (1/(1−0.996)) extra reads would need to be sequenced before detecting a new phylotype. For the hyper-variable V6 region, over 45 additional reads are needed for each new phylotype (>97.8% coverage). The coverage of the C/D-V4-0.1 samplings is still quite high, with over 150 extra reads per new phylotype discovery (>99.3% coverage), which again suggests that the substantial majority of the diversity can be captured by smaller samplings of this size (∼40,000 reads).

Diversity and evenness are more informative for describing community composition than simple phylotype richness levels. Community diversity, as reflected by the Shannon index, was highest in sample A and lowest in sample D, and is per definition generally correlated positively with the number of unique phylotypes and/or with greater community evenness. The high diversity values for V6 reads could be a consequence of higher sequence variability of the region. Thus, while the V6 amplicon sequences performed poorly for assigning taxonomies when compared with other regions, it was a better marker for capturing phylotype diversity and could therefore still be suitable for classification-independent and OTU-based (Operational Taxonomic Units) analysis.

High evenness (0≤E≤1) indicates less variation in the relative abundance of phylotypes, i.e. the number of reads per phylotypes in this case. As such, sample B contained the most even community whereas D contained the least. When ‘scaling down’ samplings for C and D the diversity index dropped somewhat, which can be expected, while there was a slight decrease in evenness for C but increase for D. This indicates that the sub-sampling was not completely uniform for all phylotypes.

### Qualitative compositional sequence analysis

**Figure 4:**
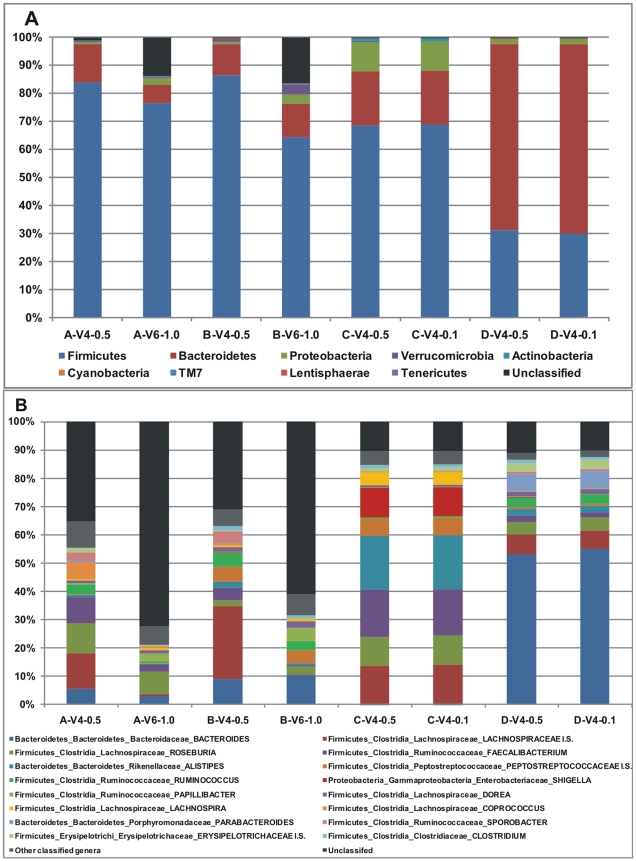
Relative phylum abundance classified with at least 50% bootstrap support (A). Relative abundance of the 16 most abundant genera classified with at least 50% bootstrap support (B). Genera are labeled according to phylum_class_family_GENUS.

While the number of subjects is too small to draw any well-founded biological conclusions, it is important to emphasize that the major aim of this study was to investigate the impact of different methods and variable regions upon the outcomes of the qualitative compositional analysis. However, there are three reasons why we still display groups of detected taxa here: Firstly, if we noticed a completely different composition for one or several of the samples, e.g. no *Firmicutes* or a vast majority of non-*Firmicutes* and non-*Bacteroidetes*, we would in the light of previous studies strongly suspect a contamination or primer problem - a quality-check in other words. Secondly, we believe that the premature freezing of sample A and B could be one reason for the small *Bacteroidetes* counts, and must therefore show these results. Lastly, by studying the different phylum and genus profiles in Figure 4 it is possible to see the similarities (or lack of) between the V6 and V4-0.5/0.1 data-sets, which is one of the aims with this study. Figure 4a shows the relative phylum abundance and Figure 4b the relative genus abundance of the most abundant genera (see Table S1 for all genera detected at bootstrap level 50%). The distributions of the major phyla (*Bacteroidetes*, *Firmicutes*, *Proteobacteria* and *Actinobacteria*) are approximately in concordance with previous human adult gut studies [15], [48] [49]. Evidently, the differently sized samplings of C and D are nearly identical by composition at both phylum and genus level. Together with the strong correlations presented in Figure 2, this suggests that equal proportions of the major taxonomic groups can be captured in smaller samplings, as for only 20% of the maximum number of reads in this case. Only a few genera were detected only by the large samplings: At most, 11 *Parabacteroides* and 4 *Akkermansia* reads were found in C-V4-0.5, but none in C-V4-0.1; while 12 *Leuconostoc* and 4 *Acinetobacter* and *Oxalobacter* reads were found in D-V4-0.5, but none in D-V4-0.1. There was, in contrast, much less agreement between the community structures as revealed by classifications of the V4 and V6 amplicon sequence data. Since the majority of V6 reads could not be classified down to genus level, this significantly hampers meaningful comparisons using that region.

As can be seen from the differences between the numbers of reads that could be classified down to genus level (153 in these samples, see Table S1), and the number of detected phylotypes/species (∼1640 at 97% V4 similarity level), most of the microbial diversity in the human gut occurs at species or strain level. This is consistent with observations of other groups [14], [50], [51] and highlights an inherent problem for obtaining high-resolution taxonomic assignments based upon variable regions of 16S rRNA gene sequences on a large scale. When investigated in another study at a much smaller scale [39], combining the three regions V2, V3 and V6 allowed assignment of all tested 110 bacterial species down to genus-level, but only a subset of these to species-level. In fact, even full-length 16S rRNA gene sequences do not always have sufficient resolving power to confidently assign species [52]. For instance, some full-length sequences obtained from different genera are more similar than 97%, while other sequences from the same species (and sometimes even within the same genome) are less similar than that [53]. However, to get an indication of how many reads can be assigned to species level (if we over-simplistically accepted a 100% match of V4/V6) we searched all confidently genus-assigned reads against the RDP database (release 10.10), from which sequences without clear species assignments had been removed, using BLAST. This resulted in 23% of all A-V4-0.5 and B-V4-0.5 reads with identical (100%) matches to known species and ∼50% of all C-V4-0.5 and D-V4-0.5 reads, but surprisingly none of the A-V6-1 and B-V6-1 reads. Hence, significant proportions of partial 16S rRNA gene sequences may not be confidently assigned to known species, for which only annotations like ‘closest relative’ and genus assignments will be possible.

### Hierarchical tree structures

To investigate an alternative to the RDP-classifier and to better visualize the compositional differences between the four communities we employed the MEGAN software [54]. MEGAN is not only another well-recognized tool for phylogenetic classification; it also bases its results on BLAST data, which is a very common method for finding nearest relatives. V4 reads from the four samples were BLAST searched against the SSU rRNA database compiled by Urich and colleagues [55], and assignment to the NCBI taxonomy was performed using the lowest common ancestor (LCA) algorithm. MEGAN uses the BLAST bit-score to assign taxonomy, as opposed to using percentage identity. As describe above, and in other OTU-based approaches, percent identities between sequences have been used as an approximate criteria for taxonomic ranks of higher-order [50], [56], [57]. Unfortunately, there is no clear correspondence between these metrics and the bit-scores that MEGAN uses; many reads with>97% identity with known taxa have lower bit-scores than hits with lower percent identities, and vice versa. Indeed, bit-scores are ultimately derived from gap scores and substitution matrices, while sequence identity simply measures proportions of identical nucleotides. We investigated three different BLAST bit-score cut-off thresholds: two previously implemented thresholds of 35 [31] and 86 [55]; and a novel threshold of 250. It was found that, at the 35 bit-score threshold, some reads (<1%) were assigned with very poor E-values and therefore could not be trusted as being valid assignments. At a bit-score cut-off of 86, more than 30% of the reads were less than 97% similar. We therefore chose a bit-score cut-off of 250, which, although still retaining reads with less than 97% identity, was determined as the best threshold by virtue of retaining the majority of true positive hits, while maintaining a minimum number of true negatives. Below we only present the results of the 250 bit-score threshold analysis.

Of the total quality-trimmed V4 reads, MEGAN assigned taxonomy to 94.9% of A-V4-0.5 reads, 99.4% of B-V4-0.5 reads, 99.1% of C-V4-0.5 reads and 93.7% of D-V4-0.5 reads at the phylum level. Reads which were not assigned taxonomy had either no hit in the database, or there was a hit but it fell below the 250 bit-score criteria for assigning taxonomy.

Analysis revealed a core gut microbiota across the four individuals; at the phylum level, this core group consisted of species from *Proteobacteria*, *Bacteroidetes*, *Firmicutes* and *Actinobacteria*, while at the genus level it consisted of, *Alistipes, Anaerostipes*, *AnaerotruncusAnaerostipes*, *Anaerotruncus*, *Bacteroides, Bifidobacterium*, *Blautia*, *Clostridium*, *Coprococcus*, *Dorea*, *Eubacterium*, *Faecalibacterium*, *Holdemania*, *Leuconostoc*, *Oscillospira*, *Peptostreptococcus*, *Roseburia*, *Ruminococcus* and *Streptococcus*. The largest groupings at phylum level were, as expected, *Firmicutes* (30–86% of total reads) and *Bacteroidetes* (10–68% of total reads). In addition, MEGAN assigned 17–37% of the reads as “unculturable organisms”.

**Figure 5:**
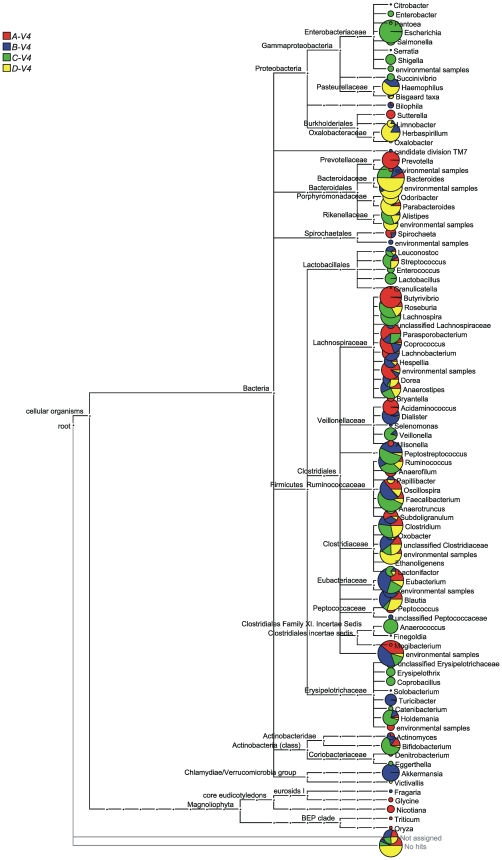
V4 amplicon sequences from the four samples assigned with BLAST and MEGAN. Pie charts display the relative abundance for each genus. ‘Not assigned’ indicates reads with BLAST hits below the cutoff value.

To compare the four communities with each other in a hierarchical way, we performed an all-against-all comparison using the MEGAN compare tool resulting in a comparison tree (Figure 5). One of the major differences observed is the low level of the genus *Bacteroides* in A-V4-0.5 and B-V4-0.5 compared with D-V4-0.5. Conversely, D-V4-0.5 has a reduced level of *Firmicutes* when compared with the others. Moreover, the C-V4-0.5 community has much higher levels of *Actinobacteria* compared with the other three datasets. Similar observations were also made from RDP-classifications, although only MEGAN assigned any of the A-V4-0.5 and B-V4-0.5 reads to phyla *Streptophyta* and *Spirochaetes*. See Table S2 for all MEGAN assignments.

**Figure 6:**
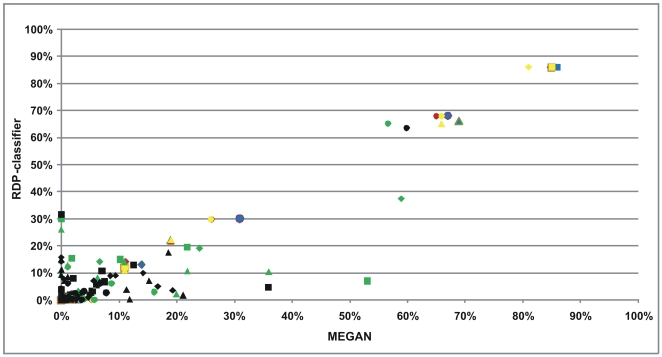
Comparisons of assignments from the RDP-classifier and MEGAN as ratios of total number of reads for each sample and taxonomic rank. Blue represents phylum, red class, yellow order, green family, and black genus. Diamonds represent sample A-V4-0.5, squares B-V4-0.5, triangles C-V4-0.5, and circles D-V4-0.5.

Direct comparisons of the RDP-classifier and MEGAN assignments show near-perfect correlations across all datasets at phylum, order and class levels with Pearson correlation coefficients of over 0.99 in each case (Figure 6 and Table S2). However, the lower correlation of B-V4-0.5 (r = 0.33) and C-V4-0.5 (r = 0.37) at the family level is due to the RDP-classifier assigning approximately ten times more reads to the *Rikenellaceae*, *Lachnospiraceae* and *Erysipelotrichaceae* families than MEGAN, whereas MEGAN assigned approximately ten times more reads to the *Clostridiaceae* than the RDP-classifier does. At the genus level, the low correlation for A-V4-0.5 (r = 0.48), B-V4-0.5 (r = 0.31) and C-V4-0.5 (r = 0.47) is due to the RDP-classifier assigning approximately ten times more reads to the *Alistipes*, *Shigella* and *Erysipelotrichaceae* genera than MEGAN, and MEGAN assigning ten times more reads to the *Clostridium* genus than the RDP-classifier. Thus, depending on organisms of interest, investigators may need to apply due caution when using either of these methods on the taxa mentioned above.

Since MEGAN assigns taxonomy based on BLAST output, it is dependent on both the BLAST algorithm and the query database, which is why it is important to use an extensive high-quality 16S rRNA gene database to optimize accuracy of the assignments. Even for strong BLAST hits, such assignments should be made with caution and are ultimately dependent on the quality of the query and subject sequences. In addition to cut-off thresholds discussed above, some factors that are likely to account for the discrepancies between RDP-classifier and MEGAN assignments are: *i)* differences between the BLAST and Bayesian algorithms; *ii)* structural differences between the Bergey and NCBI taxonomies, the latter having deeper lineages and lower rank nodes; and *iii)* incompatible training datasets and query databases. An important advantage with the MEGAN software is that it is also applicable to shotgun metagenomic sequence data, and is not limited to rRNA genes. However, the RDP-classifier is advantageous in its higher speed, as MEGAN assignments also have to include relatively slow BLAST searches against nucleotide databases. The generally high correlations between the two approaches suggest that both methods can be confidently used provided that: *i)* that the BLAST query database is sufficiently extensive and of sufficient quality; *ii)* that the bit-score threshold is adjusted to fit the required taxonomy depth, e.g. allows lower scores if genus/species assignments are the target; and *iii)* that the assignments should not be taken as absolutely definite and questionable assignments should be examined in closer detail, and confirmed or rejected using alternative assignment methods.

### Comparison of HITChip and pyrosequencing

**Figure 7:**
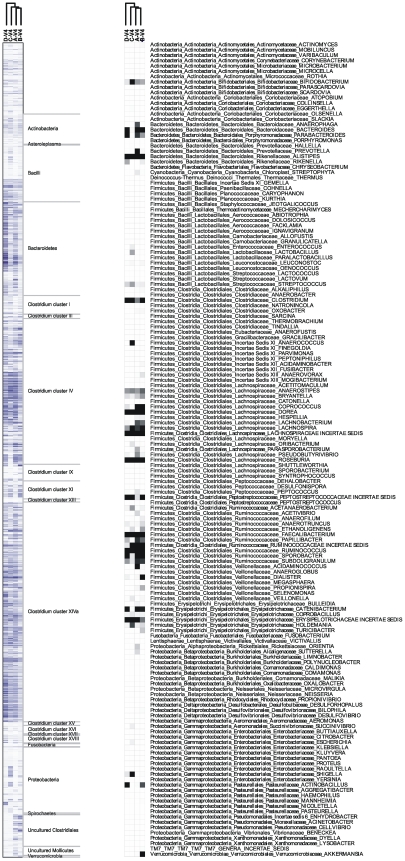
Cluster profiling of HITChip hybridization intensities (left) and number of pyrosequencing reads classified to genus-level with bootstrap support of at least 50% (right).

We compared the classification results from the pyrosequencing approach with those obtained from using the hybridization-based method employed by the HITChip. Profiling using heat maps and hierarchical clustering is a standard output of HITChip analysis [23] (Figure 7, left). For the purpose of comparison with pyrosequencing data, we clustered genus-classified reads in a similar manner (Figure 7, right). Even though they represent very different technologies and classification methods, the two phylogenetic profiles show the exact same clustering pattern, where sample A and B are the most related, followed by C and then D. This is in line with the RDP-generated data for relative genus abundance (Figure 4b), as well as the higher diversity and evenness similarity of A-V4-0.5 and B-V4-0.5. However, when varying the distance calculations, e.g. not calculating Euclidean distances and/or not using logarithmic probe intensity values, the clustering results of the two technologies were not as concordant (data not shown).

**Figure 8:**
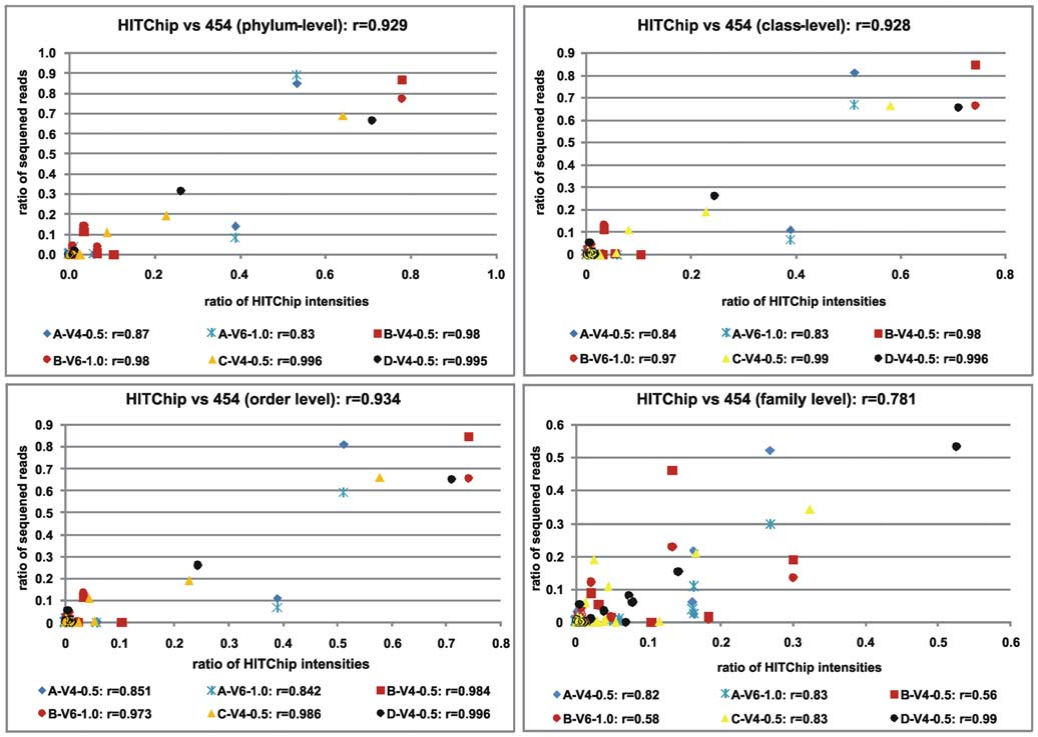
Comparisons of ratios of HITChip spot intensities and number of pyrosequencing reads for four taxonomic ranks. Pearson correlations are shown for each rank and sample.

Despite the fundamental technological differences in these approaches, it was also possible to correlate number of reads in the pyrosequencing data with probe intensity levels all the way down to family level, after the 131 HITChip taxonomic level-2 groups had been converted into RDP taxonomy. Since not all of the 131 level-2 groups were consistently at genus level, and due to one-to-many and many-to-one relationships between the two grouping schemes, it was not possible to accurately compare genus-level assignments. Figure 8 shows plots of sequence-based RDP assignments versus HITChip intensity ratios of all common taxonomic groups for the four ranks, along with Pearson correlation scores for the six different combinations of samples and variable regions. Correlations between HITChip and pyrosequencing ratios were generally good at phylum (average r = 0.94), class (0.93) and order (0.94) levels, but dropped at family level (0.77). There are two possible reasons for this: the overwhelmingly largest orders *Clostridiales* and *Bacteroidales* break down into many different families, which separately have much lower read numbers and intensities than their combinations of ranks above. As a result, these lower values drive down the correlation coefficients. Secondly, and as previously mentioned above, there are ambiguities between HITChip level-2 categories and RDP taxonomy in that a HITChip level-2 category can be either species, genus, family or more diffuse. This ‘noise’ has larger impact on family level than on lower-order ranks. The reason why most correlations using V6 sequences are lower than with V4 is that fewer V6 reads were classified with more than 50% bootstrap support.

Looking at sample-specific deviations, sample A had, for some unknown reason, much higher *Bacteroides* hybridization intensity compared to number of pyrosequencing reads. It is therefore important to employ accurate, proofreading, thermo-stable DNA polymerases, as well as temperature gradients for the PCR reactions, in order to maximize the amplification specificity [58]. Furthermore, the high number of pyrosequencing reads classified as *Shigella* genus corresponds to the *Serratia* genus using the HITChip platform. Even though they belong to the same *Enterobacteriaceae* family, this clearly highlights the issue of ambiguous classifications between the systems for some taxa, which warrants closer inspection. BLAST searches of the same V4 sequences mainly hit *E. coli* species, whereby we identified V4 as well as concatenated V1+V6 sequences from a few known *E. coli*, *Serratia* and *Shigella* species (data not shown). An all-against-all BLAST search of full-length 16S rRNA genes, as well as V1+V6 and V4 fragments of sequences from these eight species revealed that some *Serratia* species had *Shigella* and *E. coli* strains as their strongest BLAST hits in terms of higher score and percent identity, as opposed of other *Serratia* species. In addition, some *E. coli* strains had stronger hits against *Serratia* and *Shigella* than against other *E. coli* strains. Indeed, it was recently observed that the RDP-classifier cannot distinguish between *Escherichia* and *Shigella*, and by default chose *Shigella*. This will be changed to default *Escherichia* classifications in a future version of the RDP-classifier. Again, this underlines the importance of not blindly accepting all classifications of full-length or fragmented SSU rRNA sequences without closer inspections of dubious cases, irrespective of approach. Since high-throughput sequencing of partial 16S rRNA genes is becoming both more common and larger in scale, an approach targeted at classifying as many sequences as possible to species-level would be useful. Such a classifier could be based on a carefully collated database, or training-set, comprising species where sequence variation within and between close genera is known. Nevertheless, we recommend always carrying out closer inspections or complementary analysis as reported here, when uncertain, or when there is particularly high sequence similarity between taxa.

To conclude, the overall strong correlations between these two culture-independent methods indicate their robustness relative to each other, as well as their capacity for in-depth profiling of diverse microbial communities. The RDP-classifier provides fast and accurate taxonomic assignments of most pyrosequencing reads. However, for species/strain-level resolution, either longer ribosomal sequences or additional experiments are required [52], unless there are distinct and identifiable differences between the variable regions of the particular organisms of interest. We found that the V6 region was much less suitable for taxonomic classification than the V4 region, but due to its hyper-variability was a good diversity marker in being able to differentiate between more phylotypes. For single intestinal samples, diversity levels are still increasing at unprecedentedly deep sequencing levels. Nevertheless, it was possible to capture a majority of the taxa when sequencing the same samples at five times shallower coverage, in proportions equal to those resulting from the deeper half-plate samplings. This is encouraging for large-scale compositional studies where the sequencing efforts are directed towards larger number of samples, as opposed to obtaining higher resolution from fewer subjects.

## Materials and Methods

### Sample processing and sequencing

Fecal samples were collected from four elderly subjects aged 60–87 years. The Clinical Research Ethics Committee of the Cork Teaching Hospitals (CREC) granted full approval to the ELDERMET project on the 19th February 2008 (Ref: ECM 3 (a) 01/04/08). Formal written consent was obtained, on the basis of an Information Sheet/Safety Statement, following an ethics protocol that was approved by CREC, in compliance with pertaining local, national and European ethics legislation and guidelines to best practice. Subject A was male and the rest were females. Subject C had been diagnosed with ulcerative colitis, and subject D was taking an unknown antibiotic at the time of sampling. Samples from A and B were frozen at −80°C upon collection, whereas samples C and D were processed fresh from the same day as collection. DNA was extracted according to standard protocol (Qiagen, West Sussex, UK). The following universal 16S rRNA primers were used for the PCR reaction: 520F (5′-AYTGGGYDTAAAGNG-3′) and 802R (5′-TACNVGGGTATCTAATCC-3′) for the V4 region (RDP's Pyrosequencing Pipeline: http://pyro.cme.msu.edu/pyro/help.jsp); and 986F (5′-CNACGCGAAGAACCTTANC-3′) and 1027R (5′-CGACRRCCATGCANCACCT-3′) for the V6 region [11]. Barcode sequences for the V4 samples of either AGCAGAGC or AGCAGATG were attached between the 454 adaptor sequence and the forward primers. Standard PCR reaction conditions were employed for reactions with Taq polymerase – 2 mM MgCl_2_, 200 nM each primer, 200 µM dNTPs. The PCR conditions were 94°C for 50 seconds (initialization and denaturing) followed by 40°C for 30 seconds (annealing), 72°C for 60 seconds in 35 cycles (extension), and a final elongation step at 72°C for 5 minutes. Two negative control reactions containing all components, but water instead of template, were performed alongside all test reactions, and were routinely free of PCR product, demonstrating lack of contamination with post-PCR product. The optimal annealing temperature for the primers, which included 454 adapters and barcode sequences, was empirically determined by gradient PCR using control reactions with initially purified bacterial genomic DNA, and validated on fecal microbial community DNA (data not shown).

The 16S rRNA V4 and V6 amplicons were subsequently sequenced on a 454 Genome Sequencer FLX platform (Roche Diagnostics Ltd, West Sussex, UK) according to 454 protocols, one plate each for the V6 region amplicons of samples A and B, and half a plate each for the V4 region amplicons of all four samples. In addition, V4 amplicons from samples C and D were also sequenced separately on another plate as part of a pooled total of ten samples from the full-scale Eldermet project (http://eldermet.ucc.ie).

### Sequence analysis and phylogenetic classification

Raw sequencing reads were quality trimmed according to published recommendations [59] using a locally installed version of the RDP Pyrosequencing Pipeline [42] applying the following criteria: *i)* exact matches to primer sequences and barcode tags; *ii)* no ambiguous bases (Ns); *iii)* read-lengths not shorter than the main distribution (>150 bp for V4 and>60 bp for V6). For large-scale assignments into the new Bergey bacterial taxonomy [60] we used the Naïve Bayesian Classifier (RDP-classifier), which provides rapid taxonomic classifications from domain to genus of both partial and full-length rRNA gene sequences along with bootstrap-like confidence estimates [37]. Trimmed sequences with their classifications were imported into a MySQL database for efficient storage and advanced querying. Pyrosequencing reads were aligned using Infernal [61] and associated covariance models obtained from the Ribosomal Database Project Group. These were based on secondary structural information from full-length 16S rRNA genes sequences of 508 fully sequenced genomes and were further trimmed to encompass only either the V4 or V6 regions in order to increase alignment speeds. By applying the furthest neighbor approach using the Complete Linkage Clustering application of the RDP pyrosequencing pipeline, trimmed pyrosequencing sequences could be assigned to phylotype clusters of either 97% or 98% V4/V6 identity. Based on these clusters, Rarefaction curves [46], Shannon diversities [62] and Chao1 richness estimations [63] were calculated using RDP software. Good's coverage was calculated as G = 1−n/N, where n is the number of singleton phylotypes and N is the total number of sequences in the sample.

MEGAN was used for hierarchical tree constructions of the microbiota and tested as an alternative to the RDP-classifier for taxonomic assignments [64]. Based on BLAST [65] results (using default parameters with the exception –v 1 –b 1) it assigned sequences to NCBI taxonomies by employing the Lowest Common Ancestor algorithm. Bit scores were used from within MEGAN for filtering the results prior to tree construction and summarization. Following an all-against-all within MEGAN, V4 reads from all four samples were compared with each other and relative abundances displayed as pie charts in a hierarchical tree structure.

### HITChip analysis

The HITChip oligonucleotide array was designed at Wageningen University, the Netherlands [23]. Briefly, over 16,000 human intestinal full-length and partial SSU rRNA gene sequences were grouped into 1,140 unique phylotypes based on 98% or higher sequence identity. These so-called level-3 groups were also grouped into 131 genus-like level-2 groups and 27 order-like level-1 groups. Sequences from the V1 and V6 regions from each phylotype were subsequently extracted and reverse complemented, before being divided into six tiling probes that were printed on an Agilent oligonucleotide array (Agilent Technologies, Palo Alto, CA). DNA was extracted from all four fecal samples, the full-length 16S rRNA gene was amplified and further pre-processed as described by Rajilic-Stojanovic and colleagues [23], before being hybridized in duplicates onto HITChip arrays. In short, outlier probes were removed before duplicates were quantile normalized and averaged to give final intensity values for each HITChip probe, which were averaged into the 131 level-2 groups. For reproducibility, the duplicates were required to have a Pearson correlation of at least 98% (if not, they were re-hybridized). Since the grouping scheme differed significantly from the one produced by the RDP-classifier, conversion of assignments was necessary: By RDP-based classification of the initial 1,140 phylotypes with an 50% bootstrap cut-off and by using their known level-2 assignments, the 131 groups could be assigned to Bergey's taxonomy at all phylum/class/order/family levels. Spot intensities were then summarized for all taxa at every phylum/class/order/family level for each sample, but not at genus level due to lack of genus-assignments of many of the 131 groups. Ratios of total sample intensity were then compared with corresponding ratios of numbers of RDP-classified sequence reads for the same sample and taxa. Pearson coefficients were calculated as a measurement of linear correlation between sequence and intensity ratios. A heat map of median normalized HITChip intensities and associated hierarchical clustering for all four samples was also produced using logarithmic Euclidean distances followed by complete-linkage clustering. This was in turn compared with a heat map generated by Genesis [66] from pyrosequencing data, which was based on complete-linkage clustering of Euclidian distances from the numbers of genera RDP-classified with at least 50% bootstrap support.

## Supporting Information

Table S1 All 153 genera detected using the RDP-classifier with at least 50% bootstrap support.
(0.04 MB XLS)

Table S2 All MEGAN assignments of sequences from the four samples sequenced with half a pyrosequencing plate.
(0.10 MB XLS)

## References

[1] Curtis T (2007). Theory and the microbial world.. Environ Microbiol.

[2] Hopkins MJ, Sharp R, Macfarlane GT (2001). Age and disease related changes in intestinal bacterial populations assessed by cell culture, 16S rRNA abundance, and community cellular fatty acid profiles.. Gut.

[3] Woodmansey EJ (2007). Intestinal bacteria and ageing.. J Appl Microbiol.

[4] Woodmansey EJ, McMurdo ME, Macfarlane GT, Macfarlane S (2004). Comparison of compositions and metabolic activities of fecal microbiotas in young adults and in antibiotic-treated and non-antibiotic-treated elderly subjects.. Appl Environ Microbiol.

[5] Lahtinen SJ, Tammela L, Korpela J, Parhiala R, Ahokoski H (2009). Probiotics modulate the Bifidobacterium microbiota of elderly nursing home residents.. Age (Dordr).

[6] Lay C, Rigottier-Gois L, Holmstrom K, Rajilic M, Vaughan EE (2005). Colonic microbiota signatures across five northern European countries.. Appl Environ Microbiol.

[7] Zoetendal EG, Rajilic-Stojanovic M, de Vos WM (2008). High-throughput diversity and functionality analysis of the gastrointestinal tract microbiota.. Gut.

[8] Rajilic-Stojanovic M, Smidt H, de Vos WM (2007). Diversity of the human gastrointestinal tract microbiota revisited.. Environ Microbiol.

[9] Margulies M, Egholm M, Altman WE, Attiya S, Bader JS (2005). Genome sequencing in microfabricated high-density picolitre reactors.. Nature.

[10] Droege M, Hill B (2008). The Genome Sequencer FLX System—longer reads, more applications, straight forward bioinformatics and more complete data sets.. J Biotechnol.

[11] Huber JA, Mark Welch DB, Morrison HG, Huse SM, Neal PR (2007). Microbial population structures in the deep marine biosphere.. Science.

[12] Sogin ML, Morrison HG, Huber JA, Welch DM, Huse SM (2006). Microbial diversity in the deep sea and the underexplored “rare biosphere”.. Proc Natl Acad Sci U S A.

[13] Andersson AF, Lindberg M, Jakobsson H, Backhed F, Nyren P (2008). Comparative analysis of human gut microbiota by barcoded pyrosequencing.. PLoS ONE.

[14] Dethlefsen L, Huse S, Sogin ML, Relman DA (2008). The pervasive effects of an antibiotic on the human gut microbiota, as revealed by deep 16S rRNA sequencing.. PLoS Biol.

[15] Turnbaugh PJ, Hamady M, Yatsunenko T, Cantarel BL, Duncan A (2008). A core gut microbiome in obese and lean twins.. Nature.

[16] Zhang H, Dibaise JK, Zuccolo A, Kudrna D, Braidotti M (2009). Human gut microbiota in obesity and after gastric bypass.. Proc Natl Acad Sci U S A.

[17] McKenna P, Hoffmann C, Minkah N, Aye PP, Lackner A (2008). The Macaque Gut Microbiome in Health, Lentiviral Infection, and Chronic Enterocolitis.. PLoS Pathog.

[18] Roesch LF, Fulthorpe RR, Riva A, Casella G, Hadwin AK (2007). Pyrosequencing enumerates and contrasts soil microbial diversity.. Isme J.

[19] Guschin DY, Mobarry BK, Proudnikov D, Stahl DA, Rittmann BE (1997). Oligonucleotide microchips as genosensors for determinative and environmental studies in microbiology.. Appl Environ Microbiol.

[20] Palmer C, Bik EM, Digiulio DB, Relman DA, Brown PO (2007). Development of the Human Infant Intestinal Microbiota.. PLoS Biol.

[21] Harrington CR, Lucchini S, Ridgway KP, Wegmann U, Eaton TJ (2008). A short-oligonucleotide microarray that allows improved detection of gastrointestinal tract microbial communities.. BMC Microbiol.

[22] Wilson KH, Wilson WJ, Radosevich JL, DeSantis TZ, Viswanathan VS (2002). High-density microarray of small-subunit ribosomal DNA probes.. Appl Environ Microbiol.

[23] Rajilic-Stojanovic M, Heilig HG, Molenaar D, Kajander K, Surakka A (2009). Development and application of the human intestinal tract chip, a phylogenetic microarray: analysis of universally conserved phylotypes in the abundant microbiota of young and elderly adults.. Environ Microbiol.

[24] Zoetendal EG, Akkermans AD, De Vos WM (1998). Temperature gradient gel electrophoresis analysis of 16S rRNA from human fecal samples reveals stable and host-specific communities of active bacteria.. Appl Environ Microbiol.

[25] Matsuki T, Watanabe K, Fujimoto J, Takada T, Tanaka R (2004). Use of 16S rRNA gene-targeted group-specific primers for real-time PCR analysis of predominant bacteria in human feces.. Appl Environ Microbiol.

[26] Ley RE, Turnbaugh PJ, Klein S, Gordon JI (2006). Microbial ecology: human gut microbes associated with obesity.. Nature.

[27] Kajander K, Myllyluoma E, Rajilic-Stojanovic M, Kyronpalo S, Rasmussen M (2008). Clinical trial: multispecies probiotic supplementation alleviates the symptoms of irritable bowel syndrome and stabilizes intestinal microbiota.. Aliment Pharmacol Ther.

[28] Kovatcheva-Datchary P, Egert M, Maathuis A, Rajilic-Stojanovic M, de Graaf AA (2008). Linking phylogenetic identities of bacteria to starch fermentation in an in vitro model of the large intestine by RNA-based stable isotope probing.. Environ Microbiol.

[29] DeSantis TZ, Brodie EL, Moberg JP, Zubieta IX, Piceno YM (2007). High-density universal 16S rRNA microarray analysis reveals broader diversity than typical clone library when sampling the environment.. Microb Ecol.

[30] Liu Z, DeSantis TZ, Andersen GL, Knight R (2008). Accurate taxonomy assignments from 16S rRNA sequences produced by highly parallel pyrosequencers.. Nucleic Acids Res.

[31] Altschul SF, Gish W, Miller W, Myers EW, Lipman DJ (1990). Basic local alignment search tool.. J Mol Biol.

[32] DeSantis TZ, Hugenholtz P, Larsen N, Rojas M, Brodie EL (2006). Greengenes, a chimera-checked 16S rRNA gene database and workbench compatible with ARB.. Appl Environ Microbiol.

[33] DeSantis TZ,, Hugenholtz P, Keller K, Brodie EL, Larsen N (2006). NAST: a multiple sequence alignment server for comparative analysis of 16S rRNA genes.. Nucleic Acids Res.

[34] Sheneman L, Evans J, Foster JA (2006). Clearcut: a fast implementation of relaxed neighbor joining.. Bioinformatics.

[35] Kent WJ (2002). BLAT—the BLAST-like alignment tool.. Genome Res.

[36] Sundquist A, Bigdeli S, Jalili R, Druzin ML, Waller S (2007). Bacterial flora-typing with targeted, chip-based Pyrosequencing.. BMC Microbiol.

[37] Wang Q, Garrity GM, Tiedje JM, Cole JR (2007). Naive Bayesian classifier for rapid assignment of rRNA sequences into the new bacterial taxonomy.. Appl Environ Microbiol.

[38] Thompson JD, Higgins DG, Gibson TJ (1994). CLUSTAL W: improving the sensitivity of progressive multiple sequence alignment through sequence weighting, position-specific gap penalties and weight matrix choice.. Nucleic Acids Res.

[39] Chakravorty S, Helb D, Burday M, Connell N, Alland D (2007). A detailed analysis of 16S ribosomal RNA gene segments for the diagnosis of pathogenic bacteria.. J Microbiol Methods.

[40] Huse SM, Dethlefsen L, Huber JA, Welch DM, Relman DA (2008). Exploring microbial diversity and taxonomy using SSU rRNA hypervariable tag sequencing.. PLoS Genet.

[41] Edgar RC (2004). MUSCLE: multiple sequence alignment with high accuracy and high throughput.. Nucleic Acids Res.

[42] Cole JR, Wang Q, Cardenas E, Fish J, Chai B (2009). The Ribosomal Database Project: improved alignments and new tools for rRNA analysis.. Nucleic Acids Res.

[43] Gill SR, Pop M, Deboy RT, Eckburg PB, Turnbaugh PJ (2006). Metagenomic analysis of the human distal gut microbiome.. Science.

[44] Ley RE, Hamady M, Lozupone C, Turnbaugh PJ, Ramey RR (2008). Evolution of Mammals and Their Gut Microbes.. Science.

[45] Eckburg PB, Bik EM, Bernstein CN, Purdom E, Dethlefsen L (2005). Diversity of the human intestinal microbial flora.. Science.

[46] Colwell RK, Coddington JA (1994). Estimating terrestrial biodiversity through extrapolation.. Philos Trans R Soc Lond B Biol Sci.

[47] Hughes JB, Hellmann JJ, Ricketts TH, Bohannan BJ (2001). Counting the uncountable: statistical approaches to estimating microbial diversity.. Appl Environ Microbiol.

[48] Frank DN, St Amand AL, Feldman RA, Boedeker EC, Harpaz N (2007). Molecular-phylogenetic characterization of microbial community imbalances in human inflammatory bowel diseases.. Proc Natl Acad Sci U S A.

[49] Li M, Wang B, Zhang M, Rantalainen M, Wang S (2008). Symbiotic gut microbes modulate human metabolic phenotypes.. Proc Natl Acad Sci U S A.

[50] Backhed F, Ley RE, Sonnenburg JL, Peterson DA, Gordon JI (2005). Host-bacterial mutualism in the human intestine.. Science.

[51] Ley RE, Peterson DA, Gordon JI (2006). Ecological and evolutionary forces shaping microbial diversity in the human intestine.. Cell.

[52] Stackebrandt E, Ebers J (2006). Taxonomic parameters revisited: tarnished gold standards.. Microbiology Today.

[53] Wang Y, Zhang Z, Ramanan N (1997). The actinomycete Thermobispora bispora contains two distinct types of transcriptionally active 16S rRNA genes.. J Bacteriol.

[54] Huson DH, Auch AF, Qi J, Schuster SC (2007). MEGAN analysis of metagenomic data.. Genome Res.

[55] Urich T, Lanzen A, Qi J, Huson DH, Schleper C (2008). Simultaneous assessment of soil microbial community structure and function through analysis of the meta-transcriptome.. PLoS ONE.

[56] Schloss PD, Handelsman J (2005). Introducing DOTUR, a computer program for defining operational taxonomic units and estimating species richness.. Appl Environ Microbiol.

[57] Peterson DA, Frank DN, Pace NR, Gordon JI (2008). Metagenomic approaches for defining the pathogenesis of inflammatory bowel diseases.. Cell Host Microbe.

[58] Petrosino JF, Highlander S, Luna RA, Gibbs RA, Versalovic J (2009). Metagenomic pyrosequencing and microbial identification.. Clin Chem.

[59] Huse SM, Huber JA, Morrison HG, Sogin ML, Welch DM (2007). Accuracy and quality of massively parallel DNA pyrosequencing.. Genome Biol.

[60] Garrity GM, Bell JA, Lilburn TG (2004). Taxonomic outline of the procaryotes. Bergey's manual of systematic bacteriology..

[61] Nawrocki EP, Eddy SR (2007). Query-dependent banding (QDB) for faster RNA similarity searches.. PLoS Comput Biol.

[62] Gotelli NJ (2002). Ecology. Biodiversity in the scales.. Nature.

[63] Chao A, Bunge J (2002). Estimating the number of species in a stochastic abundance model.. Biometrics.

[64] Huson D, Auch A, Qi J, Schuster S (2007). MEGAN Analysis of Metagenomic Data.. Genome Research.

[65] Altschul S, Gish W, Miller W, Myers E, Lipman D (1990). Basic local alignment search tool.. Journal of Molecular Biology.

[66] Sturn A, Quackenbush J, Trajanoski Z (2002). Genesis: cluster analysis of microarray data.. Bioinformatics.

